# Modeling of MEMS Mirrors Actuated by Phase-Change Mechanism

**DOI:** 10.3390/mi8050138

**Published:** 2017-04-26

**Authors:** David Torres, Jun Zhang, Sarah Dooley, Xiaobo Tan, Nelson Sepúlveda

**Affiliations:** 1 Department of Electrical & Computer Engineering, Michigan State University, East Lansing, MI 48840, USA; torresd5@egr.msu.edu (D.T.); xbtan@egr.msu.edu (X.T.); 2 Department of Electrical & Computer Engineering, University of California, San Diego, La Jolla, CA 92093, USA; j5zhang@ucsd.edu; 3 Air Force Research Laboratory, Sensors Directorate, WP-AFB, Dayton, OH 45433, USA; sarah.dooley@us.af.mil

**Keywords:** MEMS mirrors, vanadium dioxide, phase-change materials, hysteresis, dynamic model

## Abstract

Given the multiple applications for micro-electro-mechanical system (MEMS) mirror devices, most of the research efforts are focused on improving device performance in terms of tilting angles, speed, and their integration into larger arrays or systems. The modeling of these devices is crucial for enabling a platform, in particular, by allowing for the future control of such devices. In this paper, we present the modeling of a MEMS mirror structure with four actuators driven by the phase-change of a thin film. The complexity of the device structure and the nonlinear behavior of the actuation mechanism allow for a comprehensive study that encompasses simpler electrothermal designs, thus presenting a general approach that can be adapted to most MEMS mirror designs based on this operation principle. The MEMS mirrors presented in this work are actuated by Joule heating and tested using optical techniques. Mechanical and thermal models including both pitch and roll displacements are developed by combining theoretical analysis (using both numerical and analytical tools) with experimental data and subsequently verifying with quasi-static and dynamic experiments.

## 1. Introduction

Microeletromechanical system (MEMS) mirrors are microstructures capable of redirecting an incident beam of light to a desired position. MEMS mirror devices can be characterized by their dynamic performance, degrees of freedom, size, and power consumption. The size and power consumption parameters are determined by the actuation mechanism that is implemented in the design, while the movement capability (degrees of freedom) and tilt angle amplitude are dependent on the mechanical design of the device. The speed will depend on the time response of the mechanical structure and actuation processes. The four main mechanisms implemented in MEMS mirrors are: electrostatic (ES), piezoelectric (PE) material, electromagnetic (EM) and electrothermal (ET). ES and PE use electrostatic fields for actuation—the ES commonly uses repelling/attracting forces between two plates to move the mirror platform from a resting state [1], while the PE method uses piezoelectric materials such as lead zirconate titanate (PZT) [2], where small unorganized dipoles generate material expansion and contraction upon an applied electric field. In both cases (ES and PE), mechanical forces are generated by an electric potential signal of relative large amplitude (for example: 115 V and 40 V for ES and PE, respectively), but the total power consumed by these devices is low due to the low current consumption [3,4]. The EM mechanism generates movement as the result of the force between interacting magnetic fields (Lorentz force). A possible configuration for EM consists of the interaction of a static magnetic field (created by a magnetic material) with a dynamic magnetic field (created by applying a current through a metal-trace loop inside a mirror device) [5]. The current amplitude of this mechanism can be as large as 515.17 mA for optimum performance [6]. Although the electrical actuation signals for these devices can be made very fast, their speed is ultimately determined by the dynamics of the mechanical structure. Finally, the ET mechanism uses a current to generate heat (Joule heating) on a structure, which can reach temperatures of ≈300 °C [7]. The main advantage of the ET mechanism (over ES and PE) is the much lower voltage signals required for operation. Perhaps the most common configuration for this mechanism is a bimorph structure formed by two materials (thin films) with different thermal expansion coefficients (TEC). As the temperature increases, one material will expand more than the other, generating a bending in the structure [8], concave towards the film of lower TEC. In this case, the speed of actuation will depend on the thermal dynamics of the system, making it the slowest mechanism of all for devices of similar size and thermal mass. The power consumed in this mechanism can be lower than the EM, but higher than the ES and PE, since the temperature increase depends on the amplitude of the applied current, which can be as high as 252 mA for maximum displacement [9].

A new method of actuation for MEMS mirror was presented in [10], where the conventional ET actuation mechanism (i.e., using two materials with different TEC) was replaced with a smart material, vanadium dioxide (VO_2_), which goes through a phase transition that can be induced by a gradient of temperature. VO_2_ has a reversible solid-to-solid phase transition that comes with drastic changes in the mechanical [11], electrical [12], and optical properties [13] of the material. When induced thermally, the transition of VO_2_ occurs at ≈68 °C, but this transition temperature can be reduced by doping [14] or adding extrinsic stress to the material [15,16]. The integration of VO_2_ with the MEMS mirror technology decreases the temperature required in the conventional TE mechanism, from 300 °C to 90 °C for full actuation, which lowers the total power consumed by the device. Another advantage of using VO_2_ as the actuation mechanism is the large strain energy density generated during the transition, with values higher than conventional actuation mechanisms such as thermal expansion, electrostatic, electromagnetic, and piezoelectric [17]. Furthermore, the intrinsic hysteretic behavior of VO_2_ properties (including the mechanical stress that generates deflection in VO_2_-based MEMS [18,19]) across the phase transition has been exploited to design programmable MEMS actuators [20] and resonators [21], and can be used as well to program tilting angles in MEMS mirrors. However, all of these advantages come at the cost of added nonlinear effects that make the modeling and control more complicated than other actuation mechanisms.

The modeling of MEMS mirror is a necessity to better understand the behavior of the devices. A general dynamic equation (second-order differential equation) in terms of summation of torques has been used to describe the dynamic behavior of a MEMS mirror [22,23,24]. The parameters of the equation are dependent on the mechanical structure and the actuation mechanism of the device. Different modeling and control methods have been proposed for the nonlinear hysteresis in VO_2_-based MEMS devices. Nonlinear mathematical models such as the Prandtl–Ishlinskii model [25] and the Preisach model [26] have been adopted to capture and estimate the hysteresis behaviors. Unlike the identification of the Prandtl–Ishlinskii model, which requires solving a nonlinear optimization problem, the Preisach model identification problem can be reformulated as a linear least-squares problem and solved efficiently [26]. The Preisach model is thus adopted in this work. In order to control the systems with hysteresis, feedforward control can be realized by inverting the hysteresis nonlinearity [26], and feedback control can also be implemented, where the feedback signal can be obtained based on external sensors or with self-sensing methods [27]. In self-sensing, the correlation between the electrical and mechanical properties across the transition is utilized [28].

In this work, we present a mathematical model that describes the movement of a VO_2_-based MEMS mirror. The modeling is focused on one of the four actuators of the device. First, the mechanical model of the system is derived, where the nonlinear behavior of the VO_2_ is incorporated in the model as an external force applied to the system. A Preisach model is used to capture the hysteresis behavior of the VO_2_. The parameters for the whole model are identified using simulation and experimental results. Finally, the hysteresis model is validated with a different set of experimental results including quasi-static and dynamic responses. The proposed model can be translated to other actuators of the MEMS mirror, and this work facilitates the control of the device.

## 2. Experimental Procedures

**Figure 1:**
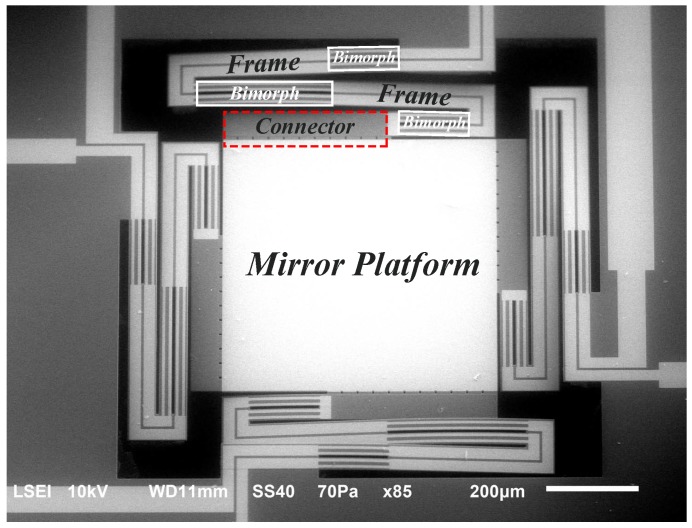
SEM image of a VO_2_-based micro-electro-mechanical system (MEMS) mirror (top view), where the different parts of an actuator leg are labeled: frame, bimorph and the connector between the mirror platform and the actuator leg.

The VO_2_-based MEMS mirror used in this paper is shown in Figure 1 and the design has been reported in [7,29,30]. The device consists of four mechanical actuators (legs) coupled with a reflective platform (mirror). Tilting of the mirror platform is achieved by individual actuation of the legs, which is independently controlled, or by actuation of all the legs using the same input signal simultaneously, which generates a piston-like movement. There are two actuation mechanisms: stress due to the thermal expansion difference of the materials forming the bimorph regions of the device, and stress generated during the phase-change transition of the VO_2_. Outside the phase transition region of VO_2_, the only active mechanism is thermal expansion, while, during the phase transition, both mechanisms exist, but the phase transition of VO_2_ dominates [26]. The generated stress is capable of bending a thin bimorph rectangular structure composed of continuous SiO_2_ (≈1.4 μm) and VO_2_ (250 nm) layers and a thin patterned metal (130 nm) layer. To increase the vertical displacement of the leg, a rigid structured (frame), composed of a thick (50 μm) layer of Si, connects the bimorphs. The transition of the VO_2_ film is induced using Joule heating, where an input current is applied through the monolithically integrated resistive heater of the leg. The metal traces are designed to have a smaller width in the bimorph parts of the leg. This is done to create a higher resistance in these regions of the heater, which increases the dissipated power and localizes the generated heat in the bimorph regions.

### 2.1. Design and Fabrication of VO_2_-Based MEMS Mirrors

The VO_2_-based MEMS mirror presented here follows the same fabrication process as in [10], but different metal layers are used to increased the yield per wafer—this is discussed in more detail in Section 2.2. Device fabrication starts with a two–inch double–sided p-type <111> polished Si wafer as the substrate with a thickness of 300 μm. A thin SiO_2_ layer (1 μm) is deposited on both sides of the wafer by plasma enhance chemical vapor deposition (PECVD) at a temperature of 300 °C. One of the SiO_2_ layers is used as a mask for the Si back-side etch, while the other (top side) forms the first layer of the bimorphs over which the VO_2_ films are deposited. A thin film from any material that can survive most chemicals used in standard MEMS processing (i.e., Si${}_{3}$N${}_{4}$) would have been an acceptable choice for the backside. However, the selection of SiO_2_ material for the top side is based on the larger mechanical actuation across the VO_2_ phase transition, which is due to the higher orientation of the VO_2_ grains with monoclinic (011)${}_{M}$ planes parallel to the substrate [20]. Although higher VO_2_ orientations are expected from crystalline substrates (i.e., quartz, sapphire), their processing represents major fabrication processing hurdles. The VO_2_ is deposited by pulse laser deposition (PLD) and patterned with dry etching using reactive ion etch (RIE), following the procedure shown in [10].

After the deposition of the VO_2_, the remaining processes are performed at temperatures lower than 250 °C to avoid any degradation in the VO_2_ due to over-oxidation of the film. A 200 nm SiO_2_ layer is grown by PECVD using a temperature of 250 °C on top of the VO_2_, for electrical isolation from the metal traces that will be deposited next. The electrical connections and resistive heaters are fabricated by depositing and patterning via lift-off layers of Cr (20 nm)/Au (110 nm), where the Cr is used as an adhesion layer between the SiO_2_ and the Au. Another 200 nm of SiO_2_ is deposited to insulate the metal traces from the ambient (air), reducing the thermal losses. This is followed by a sequence of SiO_2_ dry etch steps by RIE in order to expose the metal contacts, pattern the legs and platform of the device, and expose the Si substrate. The same SiO_2_ etching is repeated on the back-side SiO_2_ layer to expose the Si substrate. During the processing of the back-side, the top side was protected by spinning PMGA (polymethylglutarimide) resist. After processing the backside, the PMGA is removed by submerging the sample in photoresist stripper (Microposit Remover 1165). Using the SiO_2_ as a hard mask, the exposed Si layer on the backside was etched with deep reactive ion etch (DRIE). The DRIE etching is timed to remove 250 μm of the Si layer, reducing the Si substrate from 300 μm to 50 μm. The mirror structure is released by etching the remaining 50 μm of the Si substrate from the top by DRIE. Finally, to remove the Si from certain parts of the legs and create the bimorph sections, a Si isotropic etch is performed using XeF_2_ gas. This process is timed to only etch the desired parts avoiding any undesired over etch that would affect the frame regions of the legs.

### 2.2. Increasing Yield by Reducing Intrinsic Stress

In our previous work [10], we used Ti/Pt for the metallization. The rationale for the combination of these metals was to have a high-temperature metal in case it was necessary to change the order of the deposition of materials in the fabrication process flow that would require the deposition of VO_2_ after the metallization. However, the use of Ti/Pt created a low yield (≈12.5%) in the final devices, due to peeling of the metal. We thought this was due to the intrinsic stress of the evaporated Ti/Pt metal layer on SiO_2_, which could be as high as 340 MPa (compressive stress) [31,32]. In order to address the issue, in the present work, we have substituted the Ti/Pt layer with evaporated Cr/Au, which has lower intrinsic stress (250 MPa tensile stress) [33,34]. This has increased the yield to ≈75%.

### 2.3. Experimental Setup

The device is characterized by actuating only one of the four actuators and measuring the tilting angle produced by the movement. Due to symmetry of the device, the characterization of one leg can then be used to describe all the other actuators and derive the model for the entire device.

**Figure 2:**
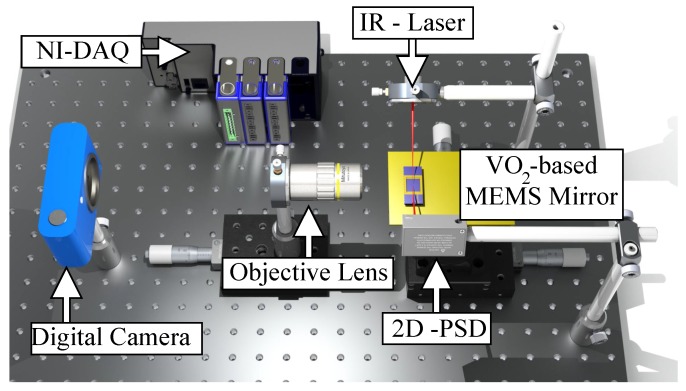
Measurement setup used for characterization of VO_2_-based MEMS mirror.

A schematic of the experimental setup is presented in Figure 2. The movement of the mirror is tracked using a laser scattering technique, where an infrared laser (λ=985 nm) with a low power is focused on the platform of the mirror and aligned with a two-dimensional (2D) position sensing detector (PSD). The 2D PSD is used to facilitate the alignment of the setup and capture any 2D movement of the device. A digital camera (Nikon 1 J1, Nikon Co., Tokyo, Japan) with an external objective lens (10× Mitutoyo Plan Apo Infinity Corrected Long WD Objective lens, Mitutoyo Corp., Kawasaki, Japan) is used for calibration purposes, by enabling the conversion of the voltage output from the PSD sensor to the tilt angle of the mirror platform. The camera is placed at the side of the sample to monitor the movement of the device. The resolution of the optical setup is 0.577 μm/pixel with a speed of 30 frames/s. The video is analyzed using Tracker Video Analysis and Modeling software tool (Version 4.94). All the electrical signals to/from the experimental setup were controlled through a virtual instrument in LabVIEW and a National Instrument data acquisition system (DAQ). The electrical current applied to the legs is controlled through the base voltage applied to a BJT NPN transistor (2n3904) in an emitter follower configuration. A resistance in series between the transistor and the actuator was used to measure the voltage and calculate the current. The transistor device was used in forward active mode.

## 3. Modeling

**Figure 3:**
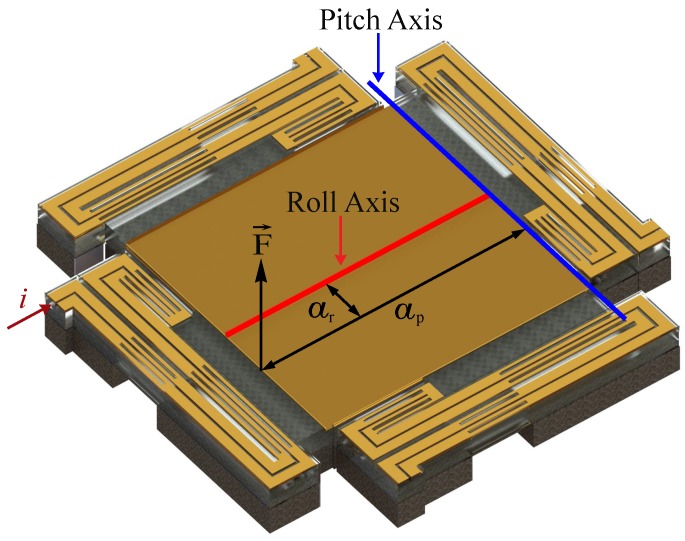
Schematic of the mirror platform showing the force ($\vec{F}$) applied by the actuated leg, and the axes of rotation for pitch and roll angles, where $a_{r}$ (115 mm) and $a_{p}$ (300 mm) are the distance between the force and each axis. In this case, the current *i* is applied to the bottom-left leg.

The connection of each mirror leg to the mirror (as shown in Figure 1) is not symmetrical along a perpendicular axis crossing the platform’s center. This offset causes the VO_2_-based MEMS mirror to have a 2D movement upon actuation. Therefore, the description and modeling of the system will involve movements along two perpendicular axes: pitch and roll. Figure 3 shows a schematic of the platform with the two axes used to describe the tilting movement of the platform. The force ($\vec{F}$) represents the actuation generated by the legs. A set of two equations (one per degree of freedom: pitch and roll) is used to model the movement of the mirror. The inclusion of the VO_2_ in the device adds a nonlinear term to the equations due to the hysteretic behavior of the material. A non-monotonic Preisach model is developed to capture the hysteresis term. The effect of the VO_2_ is included in the external force that generates actuation. The parameters for the linear part of the equation that describe the system’s mechanical response are obtained from a combination of experimental measurements and finite element method (FEM) simulations (details in Section 3.1). The coefficients of the nonlinear part of the equation are calculated from a set of experiments (details in Section 3.2).

### 3.1. Linear Model

The linear model characterizes the dynamic behavior of the system, which is approximated with a second-order differential equation

(1) $$J\ddot{\theta }+G\dot{\theta }+k\theta =\vec{T},$$

where *J* is the moment of inertia, *G* is the rotational damping coefficient, *k* is the rotational spring constant of the actuated leg, $\vec{T}$ is the external torque produced by the force $\vec{F}$, and θ is the angle of the mirror’s platform generated during actuation. The characteristic equation of the system can be derived by applying the Laplace transformation to Equation (1) and rearranging the expression to get Equation (2):

(2) $$s^{2}+s\frac{G}{J}+\frac{k}{J}=0,$$

which is in the format of a second-order characteristic equation:

(3) $$s^{2}+2\zeta \omega_{n}s+{\omega_{n}}^{2}=0,$$

where $\omega_{n}$ is the resonant frequency and ζ is the damping ratio of the actuated leg. By combining Equations (2) and (3), the moment of inertia and the rotational damping coefficient can be expressed in terms of resonant frequency and rotational spring constant:

(4) $$J=\frac{k}{{\omega_{n}}^{2}},$$

(5) $$G=2\zeta \frac{k}{\omega_{n}}.$$

The rotational spring constant (*k*) is calculated using FEM simulation of the mechanical structure, while $\omega_{n}$ and ζ are obtained from experimental results. The actuation force ($\vec{F}$) from VO_2_ is represented in $\vec{T}$ via

(6) $$\vec{T}=a\times \vec{F},$$

where *a* is the distance between the force and the axis of rotation. The generated force (*F*) from the VO_2_ can be expressed as:

(7) $$F=\Gamma [T-T_{0}],$$

where *T* is the temperature of the leg, $T_{0}$ is the ambient temperature, and Γ is the hysteresis operator describing the relationship between the generated force and the temperature of the mirror leg. The thermal process (i.e., Joule heating) can be represented as follows [35]:

$$\frac{dT(t)}{dt}=-d_{1}\left[T\left(t\right)-T_{0}\right]+d_{2}i^{2}\left(t\right),$$

$$T^{\prime }=T\left(t\right)-T_{0},$$

where $d_{1}$ and $d_{2}$ are positive coefficients related to the properties of the materials, and *i* is the input current. Applying the Laplace transform to the previous equation results in

$$sT^{\prime }=-d_{1}T^{\prime }+d_{2}i^{2}\left(s\right),$$

$$T^{\prime }=\frac{d_{2}}{s+d_{1}}i^{2},$$

$$T^{\prime }=\frac{\frac{d_{2}}{d_{1}}}{\frac{s}{d_{1}}+1}i^{2}\Rightarrow \frac{A_{T}}{\tau_{th}s+1}i^{2},$$

where $\tau_{th}$ is the thermal time constant, and $A_{T}$ is the gain of the thermal transfer function. The external force can now be expressed as:

(8) $$F=\Gamma \left[T^{\prime }\right]=\Gamma \left[\frac{A_{T}}{\tau_{th}s+1}i^{2}\right].$$

The time constant and the gain $A_{T}$ are found from experimental results. Finally, the torque generated by this force is expressed as:

(9) $$\vec{T}=a\cos(\theta )\times \Gamma \left[\frac{A_{T}}{\tau_{th}s+1}i^{2}\right],$$

which, since θ is close to zero and thus cos(θ)≈1, is simplified to

(10) $$\vec{T}=a\times \Gamma \left[\frac{A_{T}}{\tau_{th}s+1}i^{2}\right],$$

where the value *a* for the roll axis ($a_{r}$) is 115 μm and for the pitch axis ($a_{p}$) is 600 μm, as shown in Figure 3.

### 3.2. Nonlinear Model

The actuation force (*F*) is generated by two actuation mechanisms: one is the stress due to the thermal expansion difference of the materials forming the bimorph regions, and the other is the stress generated during the phase-change transition of the VO_2_. Similar to [26], a non-monotonic hysteresis model is developed:

(11) $$\Gamma \left[T^{\prime }\right]=\Gamma_{C}\left[T^{\prime }\right]+\Gamma_{E}\left(T^{\prime }\right),$$

where $\Gamma_{C}\left[T^{\prime }\right]$ is the phase transition-induced force captured by a Preisach model, and $\Gamma_{E}\left(T^{\prime }\right)$ is the differential thermal expansion-induced force modeled as a linear term.

#### 3.2.1. Phase Transition-Induced Force

The relationship between the phase transition-induced force and the temperature is monotonically hysteretic, and a Preisach model [36] is employed:

(12) $$\Gamma_{C}\left[T^{\prime }\right]\left(t\right)=\int_{P_{0}}\mu \left(\beta ,\alpha \right)\gamma_{\beta ,\alpha }\left[T^{\prime }\left(\;\cdot \;\right);\zeta_{0}\right]\left(t\right)d\beta \;d\alpha +c_{0},$$

where $P_{0}$ is the Preisach plane $P_{0}\overset{△}{=}\left\{\left(\beta ,\alpha \right):T_{\min}\leq \beta \leq \alpha \leq T_{\max}\right\}$, $\left[T_{\min},T_{\max}\right]$ define the phase transition range, μ is the density function, $\gamma_{\beta ,\alpha }$ denotes the basic hysteretic unit (hysteron), $T^{\prime }\left(\;\cdot \;\right)$ is the temperature history, $T^{\prime }\left(\eta \right)$, 0 ≤η≤t, and $c_{0}$ is a constant bias.

The hysteron is a memory-dependent operator. With the initial condition, $\zeta_{0}\in \left\{-1,1\right\}$, the output of the hysteron can be expressed as

(13) $$\gamma_{\beta ,\alpha }\left[T^{\prime }\left(\;\cdot \;\right);\zeta_{0}\right]=\left\{\begin{array}{cc} +1 & \mathrm{if}\;T^{\prime }\left(t\right)>\alpha ,\\ -1 & \mathrm{if}\;T^{\prime }\left(t\right)<\beta ,\\ \zeta_{0} & \mathrm{if}\;\beta \leq T^{\prime }\left(t\right)\leq \alpha . \end{array}\right.$$

In practical usage, the integral expression of the Preisach model is typically approximated by discretizing the weight function μ to a finite number of parameters [36,37]. The weight function is approximated as a piecewise constant function—the weight $w_{ij}$ is constant within cell (i,j), *i* = 1, 2,⋯,N; j=1,2,⋯,N-i+1, where *N* is called the discretization level and $w_{ij}$ is the model parameter. At time *n*, the output of the discretized Preisach model is expressed as

(14) $$\Gamma_{C}\left[T^{\prime }\left(n\right)\right]=\sum_{i=1}^{N}\sum_{j=1}^{N+1-i}w_{ij}s_{ij}\left(n\right)+c_{0},$$

where $w_{ij}$ is the weight for the cell (i,j) that is non-negative, and $s_{ij}\left(n\right)$ is the signed area of the cell (i,j), which is determined by the history of the temperature values up to time *n*.

The model parameters consist of the weights $\left\{w_{ij}\right\}$ and the constant bias $c_{0}$. The model identification can be reformulated as a constrained linear least-squares problem and solved efficiently with the MATLAB R2010b (Mathworks, Natick, MA, USA) command *lsqnonneg* [26,37].

#### 3.2.2. Differential Thermal Expansion-Induced Force

The differential thermal expansion-induced force is resulted from the thermal expansion difference of the VO_2_ and SiO_2_ layers. This component was modeled as a linear term and a quadratic term in previous studies [26,27]. The following linear model is adopted in this work:

(15) $$\Gamma_{E}\left(T^{\prime }\right)=-k_{0}T^{\prime },$$

where $k_{0}$ is a constant term related to thickness, modulus of elasticity, and thermal expansion coefficients of VO_2_ layer and SiO_2_ layer, and the negative term is introduced due to the fact that the thermal expansion-induced force has an opposite direction as the phase transition-induced force.

It is noted that the nonlinear model (Equations (11), (14), and (15)) can be conveniently identified with the linear least-squares method [26]. It is shown in Section 4.3 that the proposed model can accurately capture and estimate the non-monotonic hysteresis behavior of the MEMS mirror.

Finally, combining all the terms, the equations describing the movement of the mirror can be expressed as:

(16) $$J_{p}\ddot{\theta_{p}}+G_{p}\dot{\theta_{p}}+k_{p}\theta_{p}=\vec{T_{p}}=a\times \Gamma_{p}\left[\frac{A_{T}}{\tau_{{th}_{p}}s+1}i^{2}\right],$$

(17) $$J_{r}\ddot{\theta_{r}}+G_{r}\dot{\theta_{r}}+k_{r}\theta_{r}=\vec{T_{r}}=a\times \Gamma_{r}\left[\frac{A_{T}}{\tau_{{th}_{r}}s+1}i^{2}\right],$$

where the subscript *p* and *r* are references for the pitch and roll motions, the values for the linear parameters are presented in Table 3, $\Gamma_{p}$ and $\Gamma_{r}$ are the nonlinear models (Equation (11)).

## 4. Results and Discussion

### 4.1. Simulation Results

**Figure 4:**
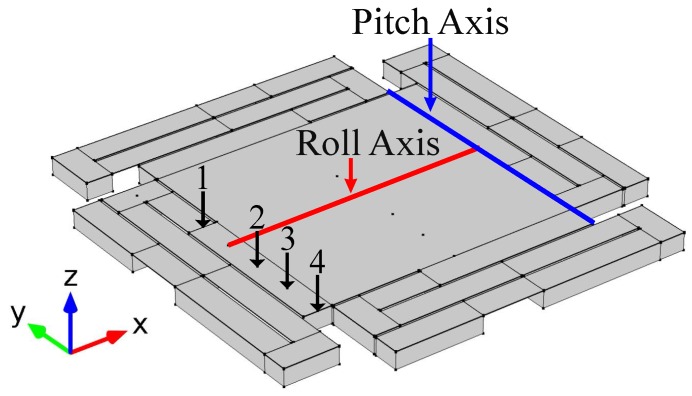
Finite element method (FEM) model schematic of the VO_2_-based MEMS mirror used to find the rotational spring constant by applying a sequence of increasing force as a point load. The force is applied at different locations (1, 2, 3 & 4) for each simulation.

An FEM model is created in COMSOL (Version 5.2a, COMSOL Inc., Burlington, MA, USA) to calculate the rotational spring constant of the leg. The parameters used for the materials on the simulation are taken from the COMSOL library and from [19] shown in Table 1. The FEM model consisted of the entire MEMS mirror structure, including the four legs, the mirror platform, and all the material layers. A force sequence of increasing magnitude is applied as a point load at the top of the leg that connects to the mirror’s platform. Four different simulations are run where the force is applied at different locations, as shown in Figure 4. The rotational spring constant at each location is extracted from the simulation results. To obtain the value of *k*, the displacement caused by the force is converted to an angle ($\theta_{sim}$) with respect to each axis by using Equation (18):

(18) $$\theta_{sim}=\sin^{-1}\frac{\left(h_{1}-h_{2}\right)}{d},$$

where $h_{1}$ and $h_{2}$ are the displacements at the point load (caused by the force) and at the axis, and *d* is the distance between the point load and the axis. The torque is then calculated by using the distance between the point load location and the corresponding axis. Finally, the torque is divided by the angle resulting in the rotational spring constant of the leg. This is done for both angles (pitch and roll) and the results are shown in Table 2.

### 4.2. MEMS Mirror Mechanical Model

**Figure 5:**
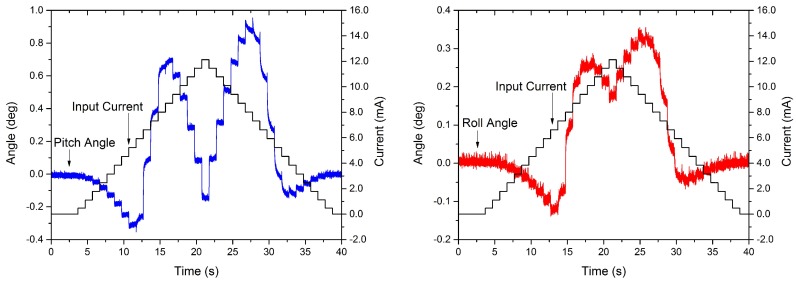
Time response measurements from actuating one leg for both variables: pitch (**left**) and roll (**right**) angles.

A set of experiments are used to characterize the mechanical response of the structure and the nonlinear behavior of the VO_2_ when actuating only one leg. Before each experiment, a pre-heating stage is performed to improve the stability and repeatability of the measurements, caused by the use of gold as the metal trace [40,41]. A similar process was performed in [7], where a sine wave was applied as the input voltage to anneal the metal layers. For the VO_2_-based MEMS mirrors in this work, the pre-heating stage consisted of applying a 12 mA to all of the actuators for a total of 10 min. An input sequence of increasing voltage steps is used to measure the thermal time response of the actuated leg. The input is applied to the base of the transistor and had increasing amplitude steps of 0.5 V, which corresponds to ≈0.7 mA (once the transistor is on). Each step is held for 1 s before the next step started. The thermal time response ($\tau_{th}$) within steps is calculated from the rise time using the following equation:

(19) $$\tau_{th}=\frac{t_{rise}}{2.2},$$

where $t_{rise}$ is the time taken for the structure to go from 10% to 90% of the output signal for one step. The results are shown in Figure 5. The thermal time response is calculated where the main dominant actuation mechanism is the thermal expansion difference of the materials forming the bimorph and not the transition of the VO_2_, the values for the pitch and roll movements are 14 ms and 14.79 ms. During the transition of the VO_2_, the system showed a pseudo-creep effect where each step took longer to reach steady state compared to outside the transition. This effect can be caused by the added stress from the legs that are not actuated. The added stress can move the transition temperature of the VO${}_{2}$, which has been observed previously in VO_2_ thin films [42,43]. Even more relevant to the present case, this effect was also observed in VO_2_-based MEMS mirrors [10], where it was found that individual leg actuation and piston-like actuation required different actuation voltages—note that, during individual actuation, the remaining mirror legs add a stress that is not present during piston-like movement. The pseudo-creep is not included in the modeling of the device, in order to focus on the fundamental thermal and mechanical dynamics in the general case, and, as verified in later experiments, the presented model (ignoring the creep effect) shows adequate capability in predicting the mirror dynamics.

**Figure 6:**
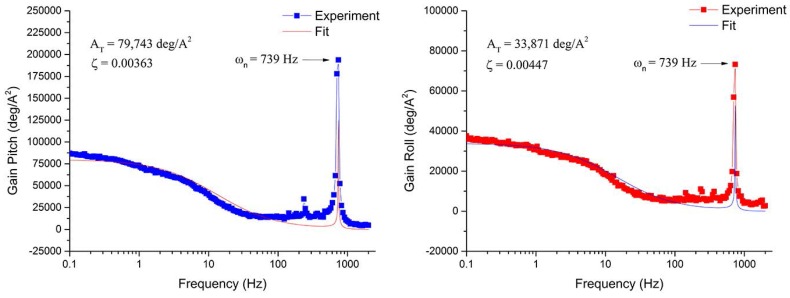
Frequency response for the actuation of one leg. A fitted curve is used to find the damping ratio (ζ) and the gain $A_{T}$. Both pitch (**left**) and roll (**right**) angles have the same resonant frequency with the value of 739 Hz.

A frequency response measurement is performed to observe the mechanical response of the system. A sine wave signal (i=1.4sin(2πft)+0.00714 mA) is applied as the input of one of the legs while the frequency is swept from 0.1 Hz to 2000 Hz. The magnitude of the displacement is measured across the whole range of frequency, and then it is divided by the magnitude of the input current. Using the software Origin Pro9.0 (OriginLab Corporation, Northampton, MA, USA), the data is fitted using the magnitude of Equation (20), with a *R*${}^{2}$ of 0.856 and 0.7797 for roll and pitch, respectively. Equation (20) is a linear approximation of the system, including the thermal and mechanical dynamics. Although the thermal response of the system in Equation (20) cannot capture the nonlinear behavior of the VO_2_, it does capture the mechanical response of the system. The values for the resonance frequency ($\omega_{n}$) and the damping ratio (ζ) for each degree of freedom are found by a curve fit, and fitting parameters are shown in Figure 6. It is worth noted that the presented curve fitting method works well at low frequencies and produces larger errors at higher frequencies above 150 Hz. This is likely due to the fact that the fitting uses linear approximation and that the mechanical couplings between each leg and between the legs and the mirror are not fully captured. The highest frequency considered in the mechanical response of the system is 10 Hz, and the model follows the experimental results fairly well in this frequency range. Analysis and modeling at higher frequencies are potential extensions to this study:

(20) $$\frac{\theta }{i^{2}}=\frac{A_{T}}{\tau s+1}\frac{{\omega_{n}}^{2}}{s^{2}+2\left(\omega_{n}\right)\zeta +{\omega_{n}}^{2}}.$$

### 4.3. Identification and Verification

#### 4.3.1. Identification

**Figure 7:**
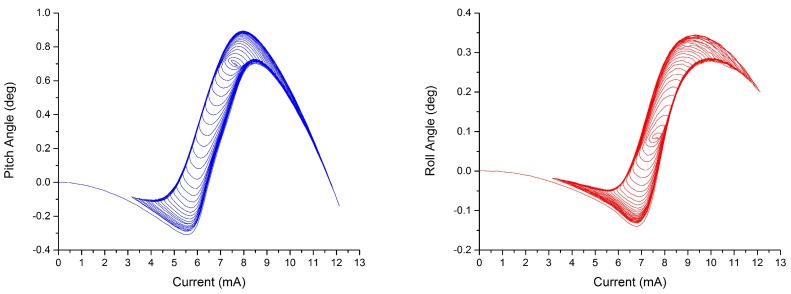
Identification plots of the pitch (**left**) and roll (**right**) angles, used to find the coefficients of the hysteresis model.

A quasi-static measurement is performed to observe the static behavior of the leg across the phase transition. A series of current steps (each held for 550 ms) are applied to one of the legs with intervals of 0.1 V. The steady-state values are obtained by averaging the last 50 ms of the pitch and roll angles. This measurement will also be used to identify the unknown variables of the hysteresis model, since it contains the minor loops of the hysteresis. The plots are shown in Figure 7. It is shown that both of the hysteresis curves exhibit non-monotonic behavior.

In order to identify the proposed model, the discretization level (*N*) of the Preisach model ($\Gamma_{C}$) is chosen to be 20. Further increasing the discretization level would increase the model complexity, but does not generate significant improvement in modeling accuracy. The root-mean-square error (RMSE) is chosen to quantify the accuracy of the model identification and verification results.

**Figure 8:**
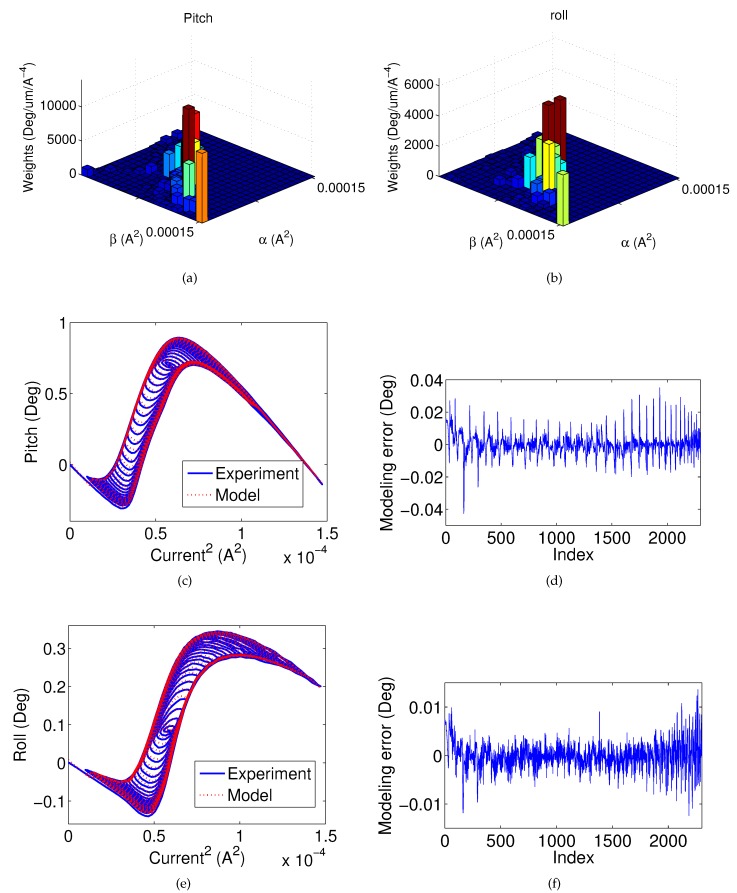
Parameters values (weights) used in the Preisach model for the (**a**) pitch and (**b**) roll; (**c**) the modeling performance; and (**d**) modeling error for the hysteresis between pitch angle and the current input; (**e**) the modeling performance; and (**f**) modeling error for the hysteresis between roll angle and the current input.

Figure 8a,b shows the identified weights of the Preisach models for the pitch and roll motions, respectively. Figure 8c shows the modeling performance for the hysteresis between the pitch angle and the current input, and Figure 8d shows the corresponding modeling error. The RMSE is 0.007 degrees. Similarly, Figure 8e–f shows the modeling performance for the hysteresis between the roll angle and the current. The RMSE is 0.003 degrees. It is shown that the proposed model can accurately capture the non-monotonic hysteresis of the MEMS mirror.

#### 4.3.2. Quasi-Static Verification

**Figure 9:**
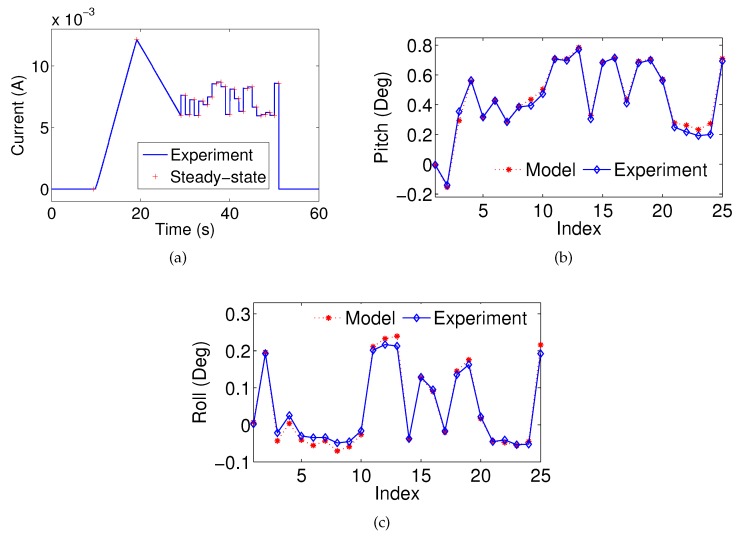
(**a**) A current step input for model verification; the measured and estimated steady-state (**b**) pitch angle; and (**c**) roll angle.

The model identification results show that the proposed model can effectively capture the hysteresis under the chosen current step input. To confirm that the model can reliably and robustly predict the pitch and roll angles under any reasonable step input, additional experiments utilizing random step inputs are conducted. A randomly-chosen current step input, as shown in Figure 9a, is applied to the MEMS mirror. Each step is held for 1 s and the corresponding steady-state pitch angle and roll angle are recorded. With the chosen current input, the pitch angle and the roll angle estimations are calculated based on identified model shown in Equations (16) and (17) with parameters provided in Figure 8a,b and Table 3. Since the quasi-static condition is considered, the derivative terms of the angles will not affect the system performance. The experimental angles are compared with the estimated values. Figure 9b shows the pitch estimation performance, and the RMSE is 0.027 degrees. Figure 9c shows the roll estimation performance, and the RMSE is 0.013 degrees. The effectiveness of the nonlinear model is confirmed.

#### 4.3.3. Frequency Verification

**Figure 10:**
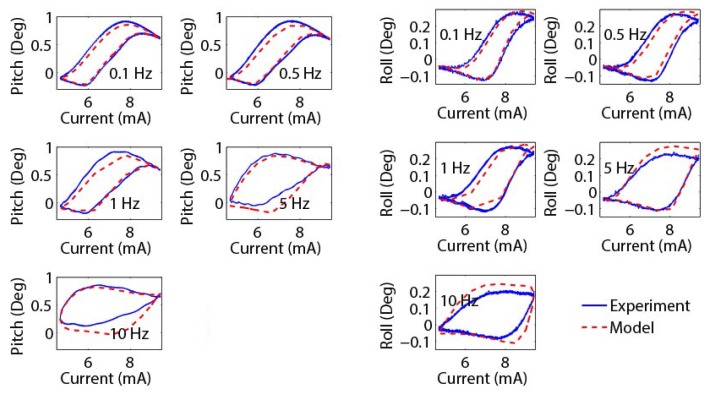
The pitch and roll angle verification performances for current inputs with different frequencies.

In order to verify that the model can effectively predict the performance of the mirror under dynamic inputs, sinusoidal current inputs with different frequencies are applied to the mirror, and the corresponding pitch angle and roll angle are recorded (Figure 10). As can be seen, the hysteresis relationships between the pitch angle and the current, and between the roll angle and the current, change under different frequencies. On average, the RMSE pitch angle estimation error is 0.074 degrees and the RMSE roll angle estimation error is 0.031 degrees. The model can capture the dynamic mirror motions reasonably well. It is noted that the estimation error becomes larger under higher frequencies, which is likely due to the mild discrepancies between the actual and calculated time response values.

#### 4.3.4. Multi-Frequency Verification

**Figure 11:**
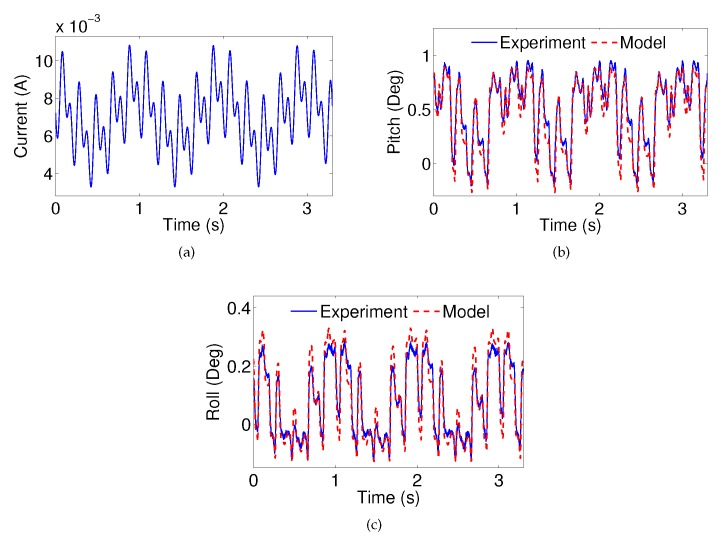
(**a**) A multi-frequency current input for model verification; the measured and estimated (**b**) pitch angle; and (**c**) roll angle.

Furthermore, the model verification for multi-frequency inputs has been conducted. The current input (1.35sin(2πt)+1.35sin(10πt)+1.35sin(20πt)+0.00705 mA), as shown in Figure 11a, is applied to the system. The model estimation performances for the pitch and roll angles are shown in Figure 11b,c, respectively. The RMSE pitch angle estimation error is 0.097 degrees and the RMSE roll angle estimation error is 0.033 degrees. The effectiveness of the proposed model for the MEMS mirror is thus further validated.

## 5. Conclusions

In this article, we have derived and verified the model for MEMS mirrors actuated by phase-change materials. The model included mechanical and thermal processes, and accounted for nonlinear behavior typically found in most phase-change materials. The approach presented here involves a combination of theoretical and experimental results, resulting in a comprehensive hybrid analysis. Although the emphasis of the present work is on MEMS mirrors actuated by phase-change materials, particularly VO_2_, the work can be extended to simpler electrothermal designs based on typical TEC difference or phase-change materials. Therefore, the present work presents a platform that can be adapted for the design of a broad scope of MEMS mirrors. Future work will focus on generalizing the presented model to other actuation modes and observing the effect of the other legs in the actuated leg and studies at higher frequencies,introducing a closed-loop control design, based on the present model, to accurately manipulate the different tilting angles of the mirror. Furthermore, future work will focus on incorporating the control system on each actuator of the VO_2_-based MEMS mirror for different purposes such as creating a 2D image or laser tracking. Additionally, a comprehensive model will be studied to incorporate the pseudo-creep behavior of the device, which is relevant for quasi-static positioning applications.

## Figures and Tables

**Table 1:** Parameters of the materials used in finite element method (FEM) simulations, where the Si, SiO_2_, and Au are obtained from the COMSOL library, while the VO_2_ properties are reported in [19].

Properties	Materials			
	Si	SiO_2_	Au	VO_2_
Density [Kg/m${}^{3}$]	2320	2200	19300	4670
Young’s Modulus [GPa]	187 [38,39]	70	70	140
Poisson Ratio	0.22	0.17	0.44	0.33

**Table 2:** Rotational spring constant from FEM simulation.

Point Load Location	Rotational Spring Constant	
	Pitch ($\times 10^{-9}\frac{N\;\cdot \;m}{\deg}$)	Roll ($\times 10^{-9}\frac{N\;\cdot \;m}{\deg}$)
1	2.29	1.219
2	2.29	1.217
3	2.29	1.217
4	2.29	1.217

**Table 3:** Coefficient values of the model.

Constant	Name and Units	Pitch ($\theta_{p}$)	Roll ($\theta_{r}$)
$A_{T}$	Gain [deg/A${}^{2}$]	79,743	33,871
$\tau_{th}$	Time response [s]	0.0014	0.001479
$\omega_{n}$	Resonant Frequency [rad/s]	4643	4643
ζ	Damping ratio	0.00363	0.00447
*J*	Moment of Inertia [Kg·m${}^{2}$]	$6.10\times 10^{-15}$	$3.23\times 10^{-15}$
*G*	Rotational Damping coefficient [N·m·s/rad]	$205.6\times 10^{-15}$	$134\times 10^{-15}$
*k*	Rotational Spring coefficient [N·m/rad]	$132\times 10^{-9}$	$69.7\times 10^{-9}$
*a*	Position of the force with respect to the axis [ mm]	600	115
$c_{0}$	Constant bias of Preisach model [ deg/mm]	0.99	0.38
$k_{0}$	Thermal expansion-induced force term [N/°C]	$1.4\times 10^{4}$	$3.8\times 10^{3}$

## References

[1] Xie H., Pan Y., Fedder G.K. (2003). A CMOS-MEMS mirror with curled-hinge comb drives. J. Microelectromech. Syst..

[2] Koh K.H., Kobayashi T., Lee C. (2011). A 2-D MEMS scanning mirror based on dynamic mixed mode excitation of a piezoelectric PZT thin film S-shaped actuator. Opt. Express.

[3] Hung A.C.L., Lai H.Y.H., Lin T.W., Fu S.G., Lu M.S.C. (2015). An electrostatically driven 2D micro-scanning mirror with capacitive sensing for projection display. Sens. Actuators A Phys..

[4] Naono T., Fujii T., Esashi M., Tanaka S. (2015). Non-resonant 2-D piezoelectric MEMS optical scanner actuated by Nb doped PZT thin film. Sens. Actuators A Phys..

[5] Yalcinkaya A., Urey H., Brown D., Montague T., Sprague R. (2006). Two-axis electromagnetic microscanner for high resolution displays. J. Microelectromech. Syst..

[6] Cho A.R., Han A., Ju S., Jeong H., Park J.H., Kim I., Bu J.U., Ji C.H. (2015). Electromagnetic biaxial microscanner with mechanical amplification at resonance. Opt. Express.

[7] Wu L., Dooley S., Watson E., McManamon P.F., Xie H. (2010). A Tip-Tilt-Piston Micromirror Array for Optical Phased Array Applications. J. Microelectromech. Syst..

[8] Jain A., Qu H., Todd S., Xie H. (2005). A thermal bimorph micromirror with large bi-directional and vertical actuation. Sens. Actuators A Phys..

[9] Samuelson S.R., Xie H. (2014). A Large Piston Displacement MEMS Mirror With Electrothermal Ladder Actuator Arrays for Ultra-Low Tilt Applications. J. Microelectromech. Syst..

[10] Torres D., Wang T., Zhang J., Zhang X., Dooley S., Tan X., Xie H., Sepúlveda N. (2016). VO_2_-Based MEMS Mirrors. J. Microelectromech. Syst..

[11] Sepulveda N., Rua A., Cabrera R., Fernández F. (2008). Young’s modulus of VO_2_ thin films as a function of temperature including insulator-to-metal transition regime. Appl. Phys. Lett..

[12] Zylbersztejn A., Mott N. (1975). Metal-insulator transition in vanadium dioxide. Phys. Rev. B.

[13] Barker A., Verleur H., Guggenheim H. (1966). Infrared optical properties of vanadium dioxide above and below the transition temperature. Phys. Rev. Lett..

[14] Mlyuka N.R., Niklasson G.A., Granqvist C.G. (2009). Mg doping of thermochromic VO_2_ films enhances the optical transmittance and decreases the metal-insulator transition temperature. Appl. Phys. Lett..

[15] Cao J., Gu Y., Fan W., Chen L., Ogletree D., Chen K., Tamura N., Kunz M., Barrett C., Seidel J. (2010). Extended mapping and exploration of the vanadium dioxide stress-temperature phase diagram. Nano Lett..

[16] Breckenfeld E., Kim H., Burgess K., Charipar N., Cheng S.F., Stroud R., Piqué A. (2017). Strain Effects in Epitaxial VO_2_ Thin Films on Columnar Buffer-Layer TiO_2_/Al_2_O_3_ Virtual Substrates. ACS Appl. Mater. Interfaces.

[17] Merced E., Tan X., Sepúlveda N. (2013). Strain energy density of VO_2_-based microactuators. Sens. Actuators A Phys..

[18] Rúa A., Fernández F.l.E., Sepúlveda N. (2010). Bending in VO_2_-coated microcantilevers suitable for thermally activated actuators. J. Appl. Phys..

[19] Cabrera R., Merced E., Sepúlveda N. (2014). Performance of Electro-Thermally Driven VO_2_-Based MEMS Actuators. J. Microelectromech. Syst..

[20] Cabrera R., Merced E., Sepúlveda N. (2013). A micro-electro-mechanical memory based on the structural phase transition of VO_2_. Phys. Status Solidi.

[21] Merced E., Cabrera R., Dávila N., Fernández F.E., Sepúlveda N. (2012). A micro-mechanical resonator with programmable frequency capability. Smart Mater. Struct..

[22] Bai Y., Yeow J.T.W., Wilson B.C. (2007). A Characteristic Study of Micromirror with Sidewall Electrodes. Int. J. Optomech..

[23] Isikman S.O., Urey H. (2009). Dynamic Modeling of Soft Magnetic Film Actuated Scanners. IEEE Trans. Magn..

[24] Han F., Wang W., Zhang X., Xie H. (2016). Modeling and Control of a Large-Stroke Electrothermal MEMS Mirror for Fourier Transform Microspectrometers. J. Microelectromech. Syst..

[25] Zhang J., Merced E., Sepúlveda N., Tan X. (2015). Optimal compression of generalized Prandtl–Ishlinskii hysteresis models. Automatica.

[26] Zhang J., Merced E., Sepúlveda N., Tan X. (2014). Modeling and Inverse Compensation of Nonmonotonic Hysteresis in VO_2_-Coated Microactuators. IEEE/ASME Trans. Mech..

[27] Zhang J., Torres D., Ebel J.L., Sepúlveda N., Tan X. (2016). A Composite Hysteresis Model in Self-Sensing Feedback Control of Fully Integrated VO_2_ Microactuators. IEEE/ASME Trans. Mech..

[28] Merced E., Torres D., Tan X., Sepúlveda N. (2015). An Electrothermally Actuated VO_2_-Based MEMS Using Self-Sensing Feedback Control. J. Microelectromech. Syst..

[29] Xie H. (2005). Vertical Displacement Device. US Patent.

[30] Wu L., Xie H. (2008). A large vertical displacement electrothermal bimorph microactuator with very small lateral shift. Sens. Actuators A Phys..

[31] Zhang L., Tsaur J., Maeda R. (2003). Residual Stress Study of SiO_2_/Pt/Pb(Zr,Ti)O_3_/Pt Multilayer Structure for Micro Electro Mechanical System Applications. Jpn. J. Appl. Phys..

[32] Matsui Y., Hiratani M., Kumagai Y., Miura H., Fujisaki Y. (1998). Thermal Stability of Pt Bottom Electrodes for Ferroelectric Capacitors. Jpn. J. Appl. Phys..

[33] Kinbara A., Haraki H. (1965). Internal Stress of Evaporated Thin Gold Films. Jpn. J. Appl. Phys..

[34] Kebabi B., Malek C., Ladan F. (1990). Stress and microstructure relationships in gold thin films. Vacuum.

[35] Leo D.J. (2007). Engineering Analysis of Smart Material Systems.

[36] Mayergoyz I. (1991). Mathematical Models of Hysteresis and Their Applications.

[37] Tan X., Baras J. (2004). Modeling and control of hysteresis in magnetostrictive actuators. Automatica.

[38] Nye J. (1985). Physical Properties of Crystals: Their Representation by Tensors and Matrices.

[39] Wortman J.J., Evans R.A. (1965). Young’s Modulus, Shear Modulus, and Poisson’s Ratio in Silicon and Germanium. J. Appl. Phys..

[40] Gall K., Dunn M.L., Zhang Y., Corff B.A. (2004). Thermal cycling response of layered gold/polysilicon MEMS structures. Mech. Mater..

[41] Gall K., West N., Spark K., Dunn M.L., Finch D.S. (2004). Creep of thin film Au on bimaterial Au/Si microcantilevers. Acta Mater..

[42] Tsai K.Y., Chin T.S., Shieh H.P.D., Ma C.H. (2004). Effect of as-deposited residual stress on transition temperatures of VO_2_ thin films. J. Mater. Res..

[43] Case F.C. (1984). Modifications in the phase transition properties of predeposited VO_2_ films. J. Vac. Sci. Technol. A Vac. Surf. Films.

